# The NAS Perchlorate Review: Is the RfD Acceptable?

**DOI:** 10.1289/ehp.113-a729

**Published:** 2005-11

**Authors:** Joan Strawson, Michael L. Dourson, Qiyu (Jay) Zhao

**Affiliations:** Toxicology Excellence for Risk Assessment, Cincinnati, Ohio, E-mail: dourson@tera.org

Risk assessors should always carefully evaluate whether a given reference dose (RfD) is the most appropriate choice for assessing risk. In the case of perchlorate, Ginsberg and Rice (2005) suggested that the RfD proposed by the National Research Council (NRC) is inappropriate because the NRC did not thoroughly evaluate the underlying science. However, we suggest that the NRC RfD is inappropriate because of the NRC’s “unconventional” approach.

In contrast to Ginsberg and Rice (2005), we applaud the insightful and conclusive discussion of the science of perchlorate and the thyroid by the NRC (2005). The NRC concluded that human studies are the most relevant for risk assessment, and that the thyroid has a remarkable ability to compensate for iodine deficiency, so that hypothyroidism would be the first observed adverse effect. By definition, this is perchlorate’s critical effect (Faustman and Omenn 2001), although U.S. Environmental Protection Agency (EPA) methods allow for the use of a known and immediate precursor (the choice of immediate precursor is based on practice of using the highest no observed adverse effect level (NOAEL) of the critical effect and is codified in several places (e.g., Barnes and Dourson 1988, p. 473). The NRC also concluded that in healthy adults the perchlorate dose required to cause hypothyroidism would be > 0.4 mg/kg-day.

In risk assessment parlance, this dose would be a NOAEL of the critical effect. The practice of risk assessment allows us to draw conclusions about public health in the absence of observable data and in the presence of scientific uncertainty. The traditional practice of developing RfD, a dose–response part of risk assessment (Barnes and Dourson 1988), would suggest two possible approaches to developing an RfD from the perchlorate data. The first would be to use the NOAEL of the critical effect from an adult population and apply uncertainty factors to account for sensitive populations and for lack of precision in defining a NOAEL. The second approach would be to use the NOAEL of an immediate precursor effect in a sensitive population and apply appropriate uncertainty factors.

Using the first approach with the NRC NOAEL, the RfD would lie in the range of 0.04–0.004 mg/kg-day depending on the choice of uncertainty factor. Using the second approach, a NOAEL of 0.005 mg/kg-day (Gibbs et al. 2004) can be identified from thyroid hormone and goiter data in a sensitive population. The RfD based on this approach would lie near the value of 0.002 mg/kg-day proposed by Strawson et al. (2004).

In contrast, the approach the NRC actually used was a nonstandard approach for developing an RfD based on the inhibition of iodine uptake, a distant precursor to the critical effect. This nonstandard approach yields a safe dose, but it is not an RfD, by definition, because, according to the NRC’s own scheme, it is not based on the critical effect or its known and immediate precursor.

We continue to advocate that the best risk assessment approach for perchlorate is to use data collected from sensitive populations such as children and, in particular, the published and ongoing work in Chile. This is consistent with the NRC’s conclusion that the data from Chile could be considered in the evaluation of the U.S. experience with perchlorate in drinking water (NRC 2005). Specifically, the Chilean experience (Crump et al. 2000; Tellez et al. 2005) can be used to help frame the public debate in the United States, which suggests perchlorate water standards as low as 1 ppb. In Chile, perchlorate water concentrations of 100–120 ppb do not result in an exposure that would inhibit iodine uptake inhibition in adults. In fact, these concentrations have not caused any adverse effects in pregnant women, neonates, or older children exposed chronically. Following traditional RfD methods and using data from a sensitive human population results in an RfD that can be used with high confidence in the United States.
